# Magnesium and Micro-Elements in Older Persons

**DOI:** 10.3390/nu13030847

**Published:** 2021-03-05

**Authors:** Nicola Veronese, Mario Barbagallo

**Affiliations:** Geriatrics Section, Department of Internal Medicine, University of Palermo, 90127 Palermo, Italy; mario.barbagallo@unipa.it

Macro- and micro-element deficiencies are widely diffused in older people. The deficiency of these elements in older people is often attributable to malnutrition, even if other medical conditions (such as gastrointestinal problem) or non-medical conditions (such as polypharmacy) can lead to these deficiencies [1]. It is estimated that malnutrition is present in 1.3–47.8% of older people living in the community, being higher in other settings and in low-middle income countries [2].

Malnutrition is often followed by deficiency in both macro-elements (i.e., minerals with a requested amount of at least 100 mg, e.g., calcium (Ca), sodium (Na) or Magnesium (Mg) and micro-elements (<100 mg/day, such as iron (Fe), Zinc (Zn) or Selenio (Se). Moreover, the scarcity of trace elements, such as Chromium (Cr+++), Silicium (Si), or Vanadium (V), could be present in malnourished older people. 

At the same time, for several years, it was assumed that malabsorption of both macro- and micro-elements was a common problem among older people [3], but increasing literature has suggested that it is not ever true, since older persons who malabsorb macronutrients often do so because of a disease (such as cancer or gastrointestinal problems), not because of age itself [3].

From an epidemiological point of view, the limited introduction of macro- and micro-elements is traditionally associated with a wide spectrum of medical conditions common in geriatric medicine, especially metabolic and cardiovascular diseases [4], dementia [5], frailty [6], sarcopenia [7] and, finally, mortality [8]. 

For these reasons, in this Special Issue entitled Magnesium and Micro-Elements in Older Persons and published in Nutrients, we decided to report the state-of-the-art regarding the deficiency of both Mg and micro-elements and the consequences in terms of higher risk of certain diseases in geriatric medicine. 

In particular, the interest in Mg is due to several reasons. First, Mg is sometimes defined as “the forgotten electrolyte” [9], since it is less frequently required in our patients than other similar elements. Despite this, as also shown in our Special Issue, poor Mg status is associated with several negative outcomes in older people. For example, novel data on Mg and dementia in more than 10,000 older participants followed-up for about 25 years were reported, showing that low midlife serum Mg is associated with increased risk of incident dementia, independently from several confounder factors [10]. Other authors have explored the importance to supplement Mg for increasing vitamin D levels in post-menopausal women [11] and for improving several cardiovascular biomarkers [12]. These novel findings could partly explain the role of Mg in improving outcomes in infectious diseases [13] and for improving hypertension [14], one of the most common condition in older subjects, as discussed in two reviews. Furthermore, the correct assessment of Mg status, particularly in older people, is often problematic. Serum Mg, in fact, could be not considered as a good proxy of Mg deposits in human bodies, since it is poorly correlating with intracellular Mg [15]. Other laboratory assessments are expensive and difficult to realize in daily clinical practice, particularly in older subjects [16]. It is important to note that about 2/3 of older people did not consume enough amounts of this micronutrient [17] and as previously mentioned, Mg deficiency is associated with several conditions in geriatric medicine.

In our Special Issue, we have also collected data regarding micro-elements. In our opinion, this aspect better completes the scenario of poor nutritional status in older people. Cardiovascular aspects are also covered by a systematic review that shows the importance of low sodium intake and a high potassium/sodium intake for preventing cardiovascular conditions in older people [18]. These topics are of critical importance since cardiovascular diseases are the leading cause of mortality in aged people. Furthermore, our Special Issue is completed by other studies regarding the importance of some micro-elements in sarcopenia, such as omega-3 fatty acids, and vitamins D, A, and K [19]. Finally, an interesting Review regarding iron metabolism is presented [20]. Iron deposits, in fact, can contribute to the development of inflammation, abnormal protein aggregation, and degeneration in the central nervous system that may increase the risk of several neurological disorders such as multiple sclerosis, Parkinson’s disease, Alzheimer’s disease, or stroke [20].

We hope that with this Special Issue the Reader can better understand the importance of Mg and micro-elements in healthy aging and in some medical conditions in older people, further highlighting the impelling necessity to frequently monitor the nutritional status of aged subjects, a topic often forgotten in actual geriatric medicine. 

## Data Availability

Not applicable.
